# Does coat colour influence survival? A test in a cyclic population of snowshoe hares

**DOI:** 10.1098/rspb.2022.1421

**Published:** 2023-04-12

**Authors:** Madan K. Oli, Alice J. Kenney, Rudy Boonstra, Stan Boutin, Dennis L. Murray, Michael J. L. Peers, B. Scott Gilbert, Thomas S. Jung, Vratika Chaudhary, James E. Hines, Charles J. Krebs

**Affiliations:** ^1^ Department of Wildlife Ecology and Conservation, University of Florida, Gainesville, FL 32611, USA; ^2^ School of Biological Sciences, Zoology Building, Tillydrone Avenue, University of Aberdeen, AB24 2TZ, UK; ^3^ Department of Zoology, University of British Columbia, Vancouver, Canada V6T 1Z4; ^4^ Department of Biological Sciences, University of Toronto Scarborough, Toronto, Canada M1C 1A4; ^5^ Department of Biological Sciences, and; ^6^ Department of Renewable Resources, University of Alberta, Edmonton, Alberta, Canada T6G 2E9; ^7^ Department of Biology, Trent University, Peterborough, ON, Canada K9L 1Z8; ^8^ Renewable Resources Management Program, Yukon University, Whitehorse, Yukon, Canada Y1A 5K4; ^9^ Department of Environment, Government of Yukon, Whitehorse, Yukon, Canada Y1A 2C6; ^10^ U.S. Geological Survey Eastern Ecological Science Center, Laurel, MD 20708, USA

**Keywords:** boreal ecosystem, coat colour, climate change, *Lepus americanus*, multi-state capture–mark–recapture models, phenological mismatch

## Abstract

Some mammal species inhabiting high-latitude biomes have evolved a seasonal moulting pattern that improves camouflage via white coats in winter and brown coats in summer. In many high-latitude and high-altitude areas, the duration and depth of snow cover has been substantially reduced in the last five decades. This reduction in depth and duration of snow cover may create a mismatch between coat colour and colour of the background environment, and potentially reduce the survival rate of species that depend on crypsis. We used long-term (1977–2020) field data and capture–mark–recapture models to test the hypothesis that whiteness of the coat influences winter apparent survival in a cyclic population of snowshoe hares (*Lepus americanus*) at Kluane, Yukon, Canada. Whiteness of the snowshoe hare coat in autumn declined during this study, and snowshoe hares with a greater proportion of whiteness in their coats in autumn survived better during winter. However, whiteness of the coat in spring did not affect subsequent summer survival. These results are consistent with the hypothesis that the timing of coat colour change in autumn can reduce overwinter survival. Because declines in cyclic snowshoe hare populations are strongly affected by low winter survival, the timing of coat colour change may adversely affect snowshoe hare population dynamics as climate change continues.

## 1. Introduction

Species inhabiting arctic and high-temperate habitats have evolved traits that enable them to survive winter conditions [1]. Some species migrate or hibernate during winter, and some that do not migrate or hibernate have evolved a moulting pattern that improves camouflage against snow [2,3]. Timing of hibernation, migration and moulting is often linked to factors such as ambient temperature, photoperiod, and snow initiation, duration and depth. However, anthropogenic climate change has led to significant global warming, with a concomitant reduction in the duration and depth of snow cover [4,5], potentially compromising the effectiveness of seasonal adaption [3]. The congruency of seasonal coat colour moulting to match the presence or absence of snow is particularly threatened by global warming [2]. At least 21 species of birds and mammals inhabiting arctic and subarctic habitats are known to undergo a seasonal moult that results in coat colour change, including snowshoe hares (*Lepus americanus*), Japanese hares (*Lepus brachyurus*), weasels (*Mustela erminea* and *Mustela nivalis*), arctic foxes (*Vulpes lagopus*) and several species of ptarmigan (*Lagopus* spp.) (reviewed in [3]). Some of these species may be able to deal with the timing of coat colour change either phenotypically or via evolution; however, phenotypic plasticity in moulting phenology or behavioural responses to minimize predation risk may be insufficient, and evolutionary responses too slow, to mitigate the potentially detrimental effects of rapidly changing climate [2,6]. Camouflage or other phenological changes in the timing of winter moults can have substantial demographic and evolutionary consequences [7].

The snowshoe hare is a keystone species in North American boreal forests, where it is prey to a host of species and exhibits cyclic population dynamics [8,9]. Snowshoe hares achieve high population density every 9–11 years, followed by population crash and a low phase that can last for several years [9–12]. The reproductive success of their major predators (e.g. Canada lynx, *Lynx canadensis*) is closely linked to hare abundance and thus they also cycle with hares [9]. Indeed, predation is the main proximate cause of snowshoe hare mortality, with greater than or equal to 90% of snowshoe hare deaths in some boreal forests being attributable to predation [5,13–15]. Because snowshoe hares reproduce during summer [15–17], their population always declines over winter. Predation-related mortality triggers the hare population decline and it is possible that the timing of coat colour change due to shortened winter snow duration or depth [18,19] can change predation-related mortality [6,20,21]. However, we lack an understanding of the relationship between the exact timing of coat colour change and survival in snowshoe hares beyond their southern range periphery. The potential impact of climate change further depends on whether moulting phenology can change in response to rising temperature and reduced snow duration. Although changes in moulting phenology are thought to be limited, especially in the brown–white autumn moult [2,6], we have yet to monitor coat colour in response to long-term changes in climatic conditions.

Here, we integrate 43 years of field data and capture–mark–recapture (CMR) models to investigate the long-term trend in seasonal coat colour and to test the hypothesis that the timing of coat colour change reduces snowshoe hare apparent survival (hereafter ‘survival’) in both winter and summer in Kluane, Yukon, Canada. Because seasonal coat colour species show clinal variation in timing of coat colour change depending on snow duration and depth [3,22], we expected the average percentage of white colour in snowshoe hare winter coat (hereafter, % white) to have declined during our 43 year study concomitantly with climate change. The alternative hypothesis is that changes in coat colour are fixed in local populations of hares. The camouflage function of seasonal coat colour is well established [3,23], so we hypothesized that % white coat colour influences snowshoe hare survival during the period of snowpack accumulation (autumn) and recession (spring) potentially via improved crypsis.

## 2. Material and methods

### (a) Study area

Our study was conducted near Kluane Lake in southwestern Yukon, Canada (60.954689° N, −138. 034778° W). Vegetation in this region is boreal forest dominated by white spruce (*Picea glauca*), with small areas of balsam poplar (*Populus balsamifera*) and trembling aspen (*Populus tremuloides*). Climate is cold, with mean monthly temperature falling below −20°C during the winter months. The study area is typically under snow cover for about 7–8 months from October to May. A detailed description of the study area is given by Krebs *et al*. [9]. The mean winter temperature in our study area has been increasing at the rate of 0.75°C per decade, which has affected snow depth and duration in complex ways (electronic supplementary material, figure S1; also see [24,25]).

### (b) Study species

Snowshoe hares are distributed across North American boreal forests [26], where they are one of the most important herbivores in terms of biomass and impact across trophic levels [27]. Their coat colour is pure white in winter but rusty brown during summer. Average lifespan is about 1 year but they can live up to 7 years [13,15,28,29]. At Kluane, greater than or equal to 90% of hares die because they are killed by predators [15,28]. Hares breed from May to September; females typically produce up to three (and occasionally four) litters per breeding season, with the first litters being born in May [16,30] and some females still weaning young in September. Mean litter size varies between 3.2 and 6.9 leverets, depending on litter order and cyclic phase [15,16,31]. Juveniles of both sexes disperse predominantly between 30 and 60 days of age; leverets typically disperse by September [15,17]. Dispersal rate is generally low [32] and cannot explain the substantial (sevenfold) seasonal difference in recruitment. Reproductive parameters typically show cyclic variation [15,16,30], but there is no evidence that dispersal is phase-specific [32]. During summer, hares consume forbs, grasses, leaves and some woody browse, but their winter diet in Kluane consists primarily of woody browse, specifically the twigs of dwarf birch (*Betula glandulosa*) and willow (*Salix glauca*) [28].

### (c) Field methods

We captured snowshoe hares using Tomahawk live traps (Tomahawk Live Trap Co., Tomahawk, WI, USA) located on 10 × 10 or 20 × 20 grids with 30 m spacing between traps. Field methods are described in detail elsewhere [15,33,34]. Each hare received an eartag (Monel no. 3; National Band and Tag Co., Newport, KY, USA) at first capture, and the identity of previously marked hares was recorded at subsequent captures. For each captured hare, the percentage of the coat that was white (hereafter, % white) was visually estimated at 5% increments using a standardized protocol. Because all individuals who live-trapped snowshoe hares used the same coat colour scoring sheet, visual estimates of % white were generally consistent across observers. However, some variation among individuals was inevitable, but this variation was insubstantial; replicate estimates of % white by two different observers of the same hare caught on two consecutive days differed by 6% (*n* = 563 hares, s.e. = 0.26%). Live trapping began in autumn 1977 and continued until autumn 2021. We considered two sampling occasions each year, autumn (hares captured during 1 September–31 October) and spring (1 March–30 April). This definition of season was chosen to ensure reasonable sample size and also because prior to September all hares are completely brown and prior to March all hares are completely white. When a hare was encountered more than or equal to two times within a sampling occasion, we calculated the mean value for % white.

### (d) Capture–mark–recapture analysis

Quantitatively assessing the influence of % white on hare survival necessitates information on % white and on the fate of the animals. However, most hares are not encountered on every sampling occasion; consequently, complete data on % white and fate are not available for each individual. Thus, we used two complementary modelling approaches to assess the influence of coat colour on the survival of snowshoe hares. First, we used the Cormack–Jolly–Seber (CJS) model to model the influence of coat colour (% white; measured during the sampling occasion when the hare was captured and ear-tagged for the first time, and coat colour quantified) on hare survival during the first interval after release. Based on previous studies [11,35], we know that snowshoe hare capture probability (*p*) varies depending on cycle number (cycle that peaked in 1980 = cycle 1 and the recent cycle that peaked in 2016–2017 = cycle 5, treated as a categorical variable), season (spring or autumn) and phase (increase, peak, decline and low) of the population cycle. Thus, we allowed *p* to be influenced by additive and interactive effects of cycle number, season and phase of the population cycle. Similarly, apparent survival probability (*φ*, the probability that an individual that is in the population at time *t* is alive and in the sampled population at time *t* + 1) was allowed to be different between the first interval after the initial release, and subsequent intervals, and to be influenced by additive and interactive effects of season and phase of the population cycle. Finally, we allowed snowshoe hare coat colour to influence *φ* interactively with the aforementioned covariates because we expected coat colour to affect summer and winter survival differently. We limited inferences regarding the influence of coat colour on survival during the first interval after release, because coat colour measurements obtained during the initial capture were used in our analyses.

Second, we used a multi-state CMR modelling framework for assessing the influence of coat colour on state-specific survival [36–38]. At each encounter, a snowshoe hare was placed in one of the three coat colour ‘states’ based on % white: A (% white less than or equal to 33%); B (% white 34–66%) and C (% white greater than 66%). We then used multi-state CMR models to estimate state-specific apparent survival (*S_k_*: the probability that a hare in state *k* survives from sampling occasion *t* to *t* + 1 and remains in the study area), capture probability (*p_k_*: the probability that a hare in state *k* is captured if it is alive and present in the study area) and transition probability (*ψ_jk_*: probability that a hare in state *j* at sampling occasion *t* transitions to state *k* at sampling occasion *t* + 1, conditional on survival). We allowed *S_k_* to be affected by singular, additive and interactive effect of phase of the cycle (increase, peak, decline and low), and season (autumn and spring); we considered only the interactive effect of season and state, because we expected coat colour states to affect summer survival and winter survival differently. We allowed *p_k_* to be affected by singular, additive and interactive effects of phase of the cycle, season and cycle number. The parameter *ψ* was modelled such that transitions for the interval (*t*, *t* + 1) depend only on the coat colour state at time *t*; we allowed transition to occur from each state in time *t* to all other states in time *t* + 1 without any constraints (denoted ‘*ψ*(*fromto*)’). Models also included both additive and interactive effects of season on the transition probabilities. We note that the two modelling approaches used in this study complemented each other. The CJS model allowed us to directly examine the relationship between % white and snowshoe hare survival. However, using this approach, we could only test for the relationship between the coat colour measured at the first capture and survival during the interval following the initial capture. This is because coat colour cannot be measured when the individual is not recaptured; consequently, some information is lost. We used the multi-state model because we could model the whiteness covariate probabilistically for the time periods even when the individual was not captured by creating discrete whiteness states.

Cyclic phases were defined following Keith [39], based on the finite rate of annual change (spring to spring) in snowshoe hare density (Δ*d*). Specifically, decline and increase phases were characterized by Δ*d* < 0.44 and Δ*d* > 1.89, respectively. All years between the decline and increase phases were defined as the low phase, and years between increase and the next decline were defined as the peak phase [39,40]. Snowshoe hare densities were estimated using spatially explicit CMR models [11]. We refer to survival from autumn to spring as winter (or overwinter) survival, and from spring to autumn as summer survival. Sampling intervals were specified in months; consequently, survival probabilities are monthly rates. We trapped snowshoe hares on seven grids but not all grids were trapped simultaneously or continuously through the entire 43 years of study [11,35]. To account for missing data, we fixed capture probability (in both CJS and multi-state models) to zero for grids when they were not trapped. There is no evidence of sex-specific differences in survival or capture probabilities (also see [33,41]), so sex effect was not included in the final analyses. Because timing of coat colour can be different for juvenile and adult snowshoe hares, we excluded hares weighing less than 1000 g; consequently, our inferences apply only to coat colour effects on the survival of subadult and adult snowshoe hares. We performed goodness-of-fit test of the CJS model using the program RELEASE [36] and of the multi-state CMR model using program U-CARE [42].

We implemented the CJS and multi-state CMR models using program MARK [43] version 9.0 implemented in the RMark package version 2.2.7 [44] for program R, version 4.2.0 [45]. We used an information-theoretic approach for model selection with Akaike's information criterion corrected for small sample size (AIC_c_) as a measure of model parsimony [36,46]. The effect of the aforementioned covariates was determined by comparing AIC_c_ among models with and without covariates. Models with small differences in AIC_c_ (ΔAIC_c_ ≤ 2) were considered to be indistinguishable in terms of the model likelihood.

## 3. Results

During the study period, 4494 snowshoe hares (2266 females and 2228 males) were captured a total of 8354 times, with quantification of coat colour (% white) at each capture.

**Figure 1.**
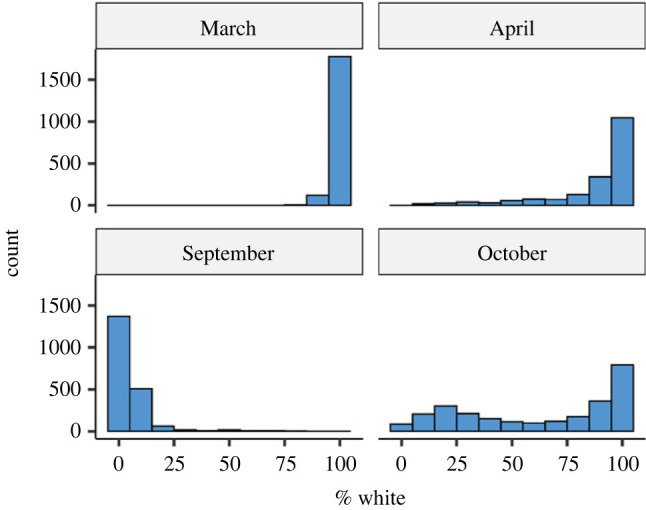
Histogram of whiteness (% white) of subadult and adult snowshoe hare (greater than or equal to 1000 g) coat colour in spring (March and April) and autumn (September and October) in Kluane, Yukon, Canada; all data combined, 1977–2020.

**Figure 2.**
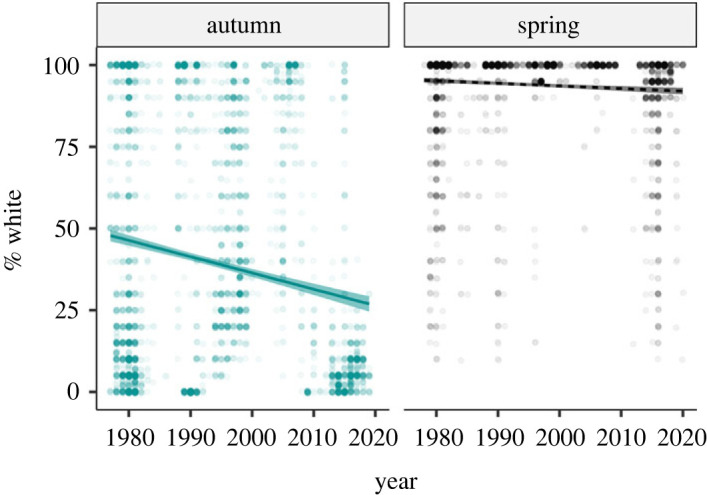
Distribution of whiteness (% white) of subadult and adult snowshoe hare (greater than 1000 g) coat colour during autumn and spring in each year of the study (1977–2020) in Yukon, Canada. Also presented is the linear regression line along with 95% confidence intervals. The darkness of the data points indicates sample size (darker = larger sample size).

**Figure 3.**
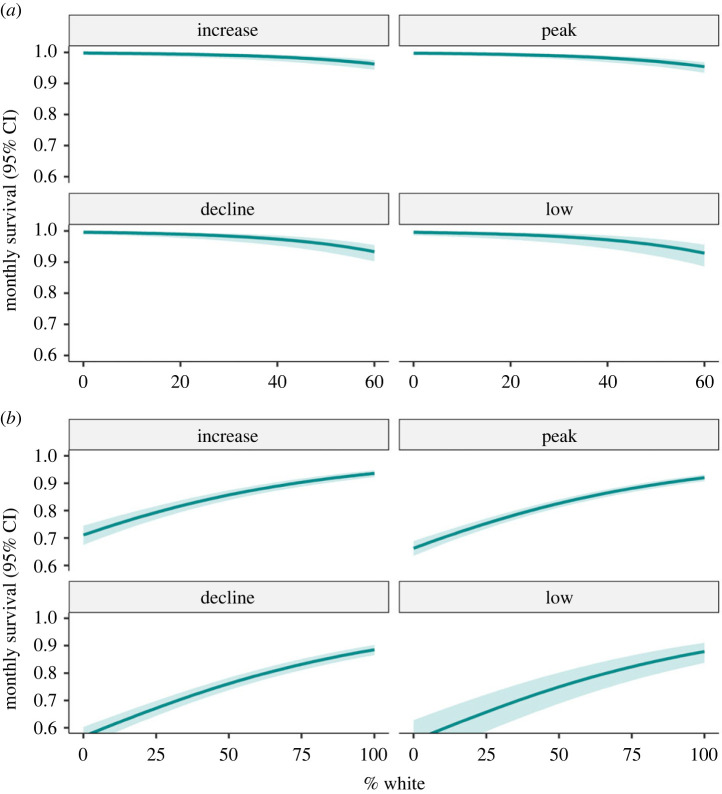
The influence of coat colour (% white) on the phase-specific monthly apparent summer (*a*) and winter (*b*) survival of subadult and adult snowshoe hares (greater than or equal to1000 g) estimated using the CJS model in Yukon, Canada, 1977–2020. Cyclic phases are: increase, peak, decline and low. Winter survival is survival from autumn to spring, and summer survival is survival from spring to autumn sampling occasions.

More hares were captured during autumn (4616 captures) than spring (3738 captures). The mean % white was substantially higher during spring (94.13 ± 0.25% s.e.) than autumn (39.47 ± 0.58%). Most snowshoe hares were pure white (100% white) in March, and pure brown at the start of September; they were in various stages of moulting in April and October (figure 1). The average whiteness of snowshoe hare coat over the defined spring and autumn seasons declined from 1977 to 2020 (figure 2), but the decline occurred at a faster rate during autumn (regression coefficient, *β* = −0.499 ± 0.041 s.e., *p* < 0.001) than in spring (*β* = −0.079 ± 0.018, *p* < 0.001). The most parsimonious CJS model included interactive effects of *two_intervals* (a variable that permits separate survival estimates for the first interval after the release and subsequent intervals), and between season and coat colour on *φ*, and three-way interactive effect of season, cyclic phases and cycle number on *p*; however, this model differed from its closest rival by ΔAICc less than 1 (table 1*a*). The RELEASE goodness of fit test indicated overdispersion (*χ*^2^ = 324.222, d.f. = 202, $\hat{c}=1.60$); thus, we made quasi-likelihood adjustments using the estimated $\hat{c}=1.60$. The top two models remained unchanged, but the model with fewer parameters was favoured after the quasi-likelihood adjustment. Virtually all well-supported models for *φ* included additive and interactive effects of coat colour and season (table 1*b*). Across all phases, winter survival was positively influenced by the whiteness of the snowshoe hare coat measured in the autumn of first capture. However, coat colour in spring had no discernible effect on apparent survival of snowshoe hares during summer (figure 3).

**Table 1.** Model comparison statistics testing for the effect of coat colour (coat.mean) and other covariates on capture probability (*p*) and apparent survival (*φ*) for snowshoe hares using (*a*) CJS model, (*b*) CJS model after quasi-likelihood adjustment, and (*c*) multi-state CMR models, in Kluane, Yukon, Canada. Covariates are: cyclic phase (phase: increase, peak, decline, low); season (autumn or spring sampling occasions), cycle number (cycle_no; cycle peaking in 1980–1981: cycle number 1; current cycle peaking in 2016–2017: cycle number 5), *two_intervals* (a covariate that separates survival during the first interval after release and all subsequent intervals) and *fromto* (a covariate that allows transitions to differ depending on the state preceding the interval and the state just after the interval). Multi-state models were based on three coat colour states: A ≤33% white; B = 34 to 66% white; and C >66% white. Model parameters are: *p* = capture probability; *φ*, *S* = apparent survival; and *ψ* = state transition probability. For each model, we report the number of parameters (*K*), Akaike information criterion corrected for small sample size (AICc) or quasi Akaike information criterion corrected for small sample size (QAICc), difference in AICc (ΔAICc) or in QAICc (ΔQAICc), and model weight. A ‘+’ indicates additive effects of the covariates, whereas an asterisk (*) indicates both additive and interactive effects.

(*a*) CJS model (unadjusted)					
model no.	model	*K*	AICc	ΔAICc	weight
1	*φ*(phase + season ∗ coat.mean ∗ two_intervals)*p*(phase ∗ season ∗ cycle_no)	49	7922.238	0.000	0.404
2	*φ*(phase + season ∗ coat.mean + two_intervals)*p*(phase ∗ season ∗ cycle_no)	46	7922.678	0.440	0.324
3	*φ*(phase ∗ season ∗ coat.mean + two_intervals)*p*(phase ∗ season ∗ cycle_no)	55	7923.285	1.047	0.239
4	*φ*(two_intervals ∗ phase + season ∗ coat.mean)*p*(phase ∗ season ∗ cycle_no)	49	7927.311	5.074	0.032
5	*φ*(phase ∗ season ∗ coat.mean + two_intervals)*p*(phase ∗ cycle_no + season)	38	7950.543	28.306	0.000
6	*φ*(phase + season ∗ coat.mean + two_intervals)*p*(phase ∗ cycle_no + season)	29	7954.553	32.316	0.000
7	*φ*(phase + season ∗ coat.mean ∗ two_intervals)*p*(phase ∗ cycle_no + season)	32	7955.238	33.001	0.000
8	*φ*(two_intervals ∗ phase + season ∗ coat.mean)*p*(phase ∗ cycle_no + season)	32	7959.221	36.984	0.000
9	*φ*(phase + season ∗ coat.mean ∗ two_intervals)*p*(phase ∗ cycle_no)	31	7965.246	43.009	0.000
10	*φ*(phase ∗ season ∗ coat.mean + two_intervals)*p*(phase ∗ cycle_no)	37	7966.673	44.435	0.000
(*b*) CJS model (adjusted)					
model no.	model	*K*	QAICc	ΔQAICc	weight
1	*φ*(phase + season ∗ coat.mean + two_intervals)*p*(phase ∗ season ∗ cycle_no)	46	4971.001	0.000	0.661
2	*φ*(phase + season ∗ coat.mean ∗ two_intervals)*p*(phase ∗ season ∗ cycle_no)	49	4973.023	2.022	0.241
3	*φ*(two_intervals ∗ phase + season ∗ coat.mean)*p*(phase ∗ season ∗ cycle_no)	49	4976.184	5.183	0.050
4	*φ*(phase + season ∗ coat.mean + two_intervals)*p*(phase ∗ cycle_no + season)	29	4977.890	6.889	0.021
5	*φ*(phase ∗ season ∗ coat.mean + two_intervals)*p*(phase ∗ season ∗ cycle_no)	55	4978.273	7.273	0.017
6	*φ*(phase + season ∗ coat.mean ∗ two_intervals)*p*(phase ∗ cycle_no + season)	32	4980.600	9.600	0.005
7	*φ*(phase ∗ season ∗ coat.mean + two_intervals)*p*(phase ∗ cycle_no + season)	38	4982.250	11.249	0.002
8	*φ*(two_intervals ∗ phase + season ∗ coat.mean)*p*(phase ∗ cycle_no + season)	32	4983.082	12.081	0.002
9	*φ*(phase + season ∗ coat.mean + two_intervals)*p*(phase ∗ cycle_no)	28	4985.741	14.741	0.000
10	*φ*(phase + season ∗ coat.mean ∗ two_intervals)*p*(phase ∗ cycle_no)	31	4986.074	15.074	0.000
(*c*) multi-state model					
model no.	model	*K*	AICc	ΔAICc	weight
1	*S*(stratum ∗ season + phase)*p*(stratum + cycle_no)*Ψ*(fromto ∗ season)	28	979251.764	0.000	1.000
2	*S*(stratum ∗ season)*p*(stratum + cycle_no)*Ψ*(fromto ∗ season)	25	979288.573	36.809	0.000
3	*S*(stratum ∗ season + phase)*p*(stratum + phase)*Ψ*(fromto ∗ season)	27	979317.037	65.272	0.000
4	*S*(stratum)*p*(stratum + cycle_no)*Ψ*(fromto ∗ season)	22	979318.708	66.944	0.000
5	*S*(stratum ∗ season)*p*(stratum + phase)*Ψ*(fromto ∗ season)	24	979335.788	84.024	0.000
6	*S*(stratum ∗ season + phase)*p*(stratum + season)*Ψ*(fromto ∗ season)	25	979336.293	84.529	0.000
7	*S*(stratum ∗ season + phase)*p*(stratum)*Ψ*(fromto ∗ season)	24	979340.118	88.354	0.000
8	*S*(stratum ∗ season + phase)*p*(stratum + cycle_no)*Ψ*(fromto + season)	23	979350.023	98.258	0.000
9	*S*(stratum)*p*(stratum + phase)*Ψ*(fromto ∗ season)	21	979362.504	110.740	0.000
10	*S*(stratum ∗ season + phase)*p*(.)*Ψ*(fromto ∗ season)	22	979368.358	116.594	0.000

**Figure 4.**
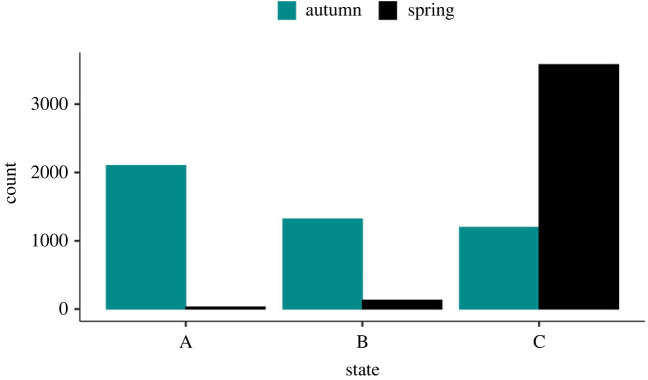
Number of captured snowshoe hares in different coat colour states during autumn and spring sampling occasions in Kluane, Yukon, Canada, 1977–2020. Coat colour states are A (% white less than or equal to 33%), B (% white 34–66%) and C (% white greater than 66%).

**Figure 5.**
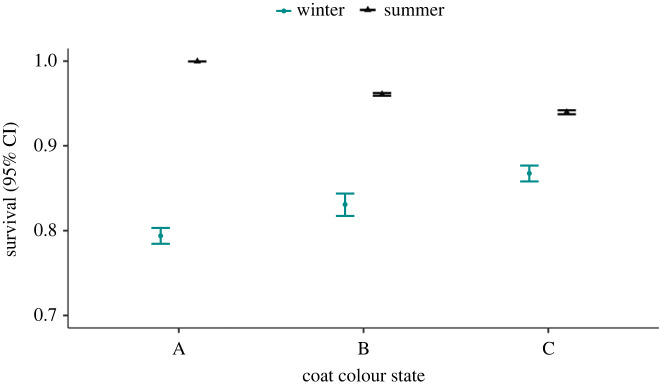
State-specific apparent monthly survival probability of snowshoe hares in each season estimated using the multi-state CMR model in Yukon, Canada, 1977–2020. Coat colour states are A (% white ≤33%), B (% white 34–66%) and C (% white greater than 66%). Summer survival for coat colour state A was not estimated owing to insufficient data.

Most snowshoe hares were in coat colour states A or B (i.e. less than or equal to 66% white) in autumn, and mostly in B or C states (greater than 33% white) in spring (figure 4). The U-CARE goodness-of-fit test provided no evidence for the lack-of-fit of the multi-state CMR model or of overdispersion (*χ*^2^ = 436.493, d.f. = 484, $\hat{c}=0.902$). The most parsimonious multi-state CMR model suggested that survival was influenced by additive and interactive effects of coat colour states and season, and an additive effect of cyclic phases, and *p* was an additive function of state and cycle number. The state-specific transition probability, *ψ*, showed seasonal variation, and depended on the state preceding the time interval and the state just after the interval (the *fromto* covariate); these probabilities also varied seasonally (table 1*c*). The top multi-state model could not estimate phase-specific summer survival rates for states A and B; thus, we examined survival estimates based on the second ranked model, which differed from the top model only in that it did not include an additive effect of phase on survival (model 2, table 1*c*). Based on this model, whiter snowshoe hares (coat colour state = C) in autumn survived substantially better during winter than less white ones (coat colour state = A or B). During summer, whiter hares had slightly lower survival than browner hares (figure 5). Estimates of capture and transition probabilities are presented in electronic supplementary material, S1, tables S1–S3, and data on average winter snow depth and average winter temperature in Kluane study site are presented in electronic supplementary material, S1, figures S1and S2).

## 4. Discussion

Coat colour in mammals generally matches the coloration of their background environments, and camouflage is thought to be the most important force driving the evolution of overall pelage coloration [23]. Crypsis may be particularly adaptive in prey species experiencing high risk of predation that rely on cryptic coloration for their survival, such that individuals that are better camouflaged against the background environment have higher survival prospects [3]. In high-altitude and high-latitude environments, the colour of the background environment changes seasonally owing to the presence of snow; seasonal coat colour is thought to have evolved to deal with seasonally changing background colour of the landscape due to snow presence (reviewed in [3]). However, global warming has substantially reduced snow duration and depth [4,19]. In our study area, winter temperature has been rising at the rate of 0.75°C per decade (electronic supplementary material, figure S1; [24,25]), and snow duration and depth are likewise declining [5,19,21], creating a potential seasonal mismatch between coat colour and colour of the background environment. This observation raises two important questions. First, has the whiteness of snowshoe hare coat colour in autumn and/or spring in North American boreal forests decreased concomitant with the rising temperature and declining snow depth and duration? Second, does whiteness of the coat influence winter or summer survival of snowshoe hare? Our goal in this study was to address these questions using long-term field data and CMR modelling approaches.

We found that whiteness (% white) of the snowshoe hare coat in autumn and spring has significantly declined over the last four decades in Canada's Yukon, but the decline has occurred at a faster rate in autumn (0.499% per year) than in spring (0.079% per year) (figure 2). Moulting in snowshoe hare is a phenotypically plastic trait, and its phenology is strongly influenced by photoperiod, local temperature and snow conditions [47]. Because of decreased snow duration and depth in our study area [5], it seems likely that the observed decline in the whiteness of snowshoe hare coat colour during transition seasons is a phenotypically plastic or an evolutionary response to reduced snow duration and depth, as hypothesized by Zimova *et al*. [47]. Previous studies have reported limited plasticity in moulting phenology, especially in the initiation of brown-to-white moult in autumn [2,6]. Our results indicate that brown-to-white autumn moult may be more labile than white-to-brown spring moult.

Consistent with findings of previous studies [5,33,35], snowshoe hare survival differed among seasons (snowshoe hares survived better during summer than winter), and across cyclic phases (survival was substantially lower during decline phase compared with the other cyclic phases). Virtually all well-supported CJS models included an interactive effect of season and coat colour, suggesting that whiteness of the coat at the time of initial capture influenced snowshoe hare survival during the first interval after release, but that the influence of coat colour at initial capture differed between summer and winter. Coat colour had no discernible impact on summer survival, but it strongly positively influenced winter survival, although the strength and pattern of this influence varied across cyclic phases (figure 3). The impact of % white coat colour on crypsis may differ between seasons.

While the CJS model provided strong evidence for the fact that whiter snowshoe hares survived better during winter, these analyses relied on coat colour measurements only at first capture. Thus, we performed a second set of analyses where we created coat colour states (state A: % white less than or equal to 33%; state B: % white 34–66%; and state C: % white greater than 66%) and applied multi-state CMR models. This approach allowed us to use coat colour measurements obtained during all captures and explicitly test the hypothesis regarding state-specific differences in survival for summer and winter seasons. In September, nearly all snowshoe hares were predominantly brown (state A), and in March, nearly all snowshoe hares were predominantly pure white (state C). However, the state distribution changed as the moulting progressed, with all three states being represented in October and April (figure 4). Consistent with our expectations as well as the results of CJS analyses, we found strong evidence of state-specific differences in winter survival, but not in summer survival.

Snowshoe hares in state C survived substantially better than those in state A during winter (figure 5). Monthly winter survival of hares in state A and state C was 79 and 83%, respectively. The importance of the difference in state-specific winter survival becomes clearer by examining differences in survival for the entire winter (overwinter survival = monthly survival raised to the sixth power for a 6-month rate). Calculated this way, overwinter survival of snowshoe hares in state A (predominantly brown) and state C (predominantly white) in the autumn during increase phase was 37 and 57%, and during decline phase it was 25 and 43%, respectively. These results are fascinating but also troubling because snowshoe hare population crashes are caused by substantial reduction in overwinter survival [12,35]. The depth and duration of snow cover is predicted to continue to decrease in boreal forests as global warming continues, which will increase the period of potential mismatch between snowshoe hare coat colour and that of the background environment. Using an experimental translocation, where snowshoe hares were translocated from their existing home ranges to a new unoccupied site recently extirpated of hares, Wilson *et al*. [48] showed that snowshoe hares in Montana, USA were three times more likely to die from predation during periods of camouflage mismatch, especially in habitats characterized by poor cover. In Wilson's study, once mismatch began, predation occurred at a high rate in the translocated habitat. In western Montana, Zimova *et al*. [20] found that snowshoe hares mismatched with the colour of their background experienced lower survival and, in the absence of adaptive responses, they predicted strong population declines by the end of the century. Recently, Peers *et al*. [5] showed that mortality risk from predation is strongly influenced by variation in snow conditions in Kluane, Yukon, such that snowshoe hares in shallow snow conditions experienced substantially higher predation risk from some mammalian predators. By using CMR analysis of long-term data, our results add to the earlier findings that changing temperature and snow conditions can have substantial negative effect on snowshoe hare survival and possibly population dynamics.

It is important here to recognize the limitations of our dataset. We have data on the % white in individual hares in autumn, and we can estimate the survival of these cohorts to the following spring. We did not follow individual hares through the winter, and we do not have any data comparing the coat colour of a hare with the colour of its background with respect to snow cover or patchiness. These more detailed studies need to be done. The strength of our dataset is that it is long-term, covering five 9- to 10-year cycles of snowshoe hares with simple data in autumn and spring of individual variation in coat colour of marked hares.

Given the findings that snowshoe hares experience high-predation risk when snow depth is low [5,48], and that the period of mismatch between hare coat colour and colour of the landscape will likely increase owing to shorter snow duration, it is possible that winter survival of snowshoe hares will decline in response to rising temperature. In the absence of plastic or evolutionary response, further reduction in snow depth and increases in camouflage mismatch could cause snowshoe hare population declines, collapse of their population cycles and possibly geographical range contraction [20,48].

It is possible that snowshoe hares may be able to deal with phenological mismatch via phenotypically plastic responses in moulting phenology or behavioural responses to minimize predation risk, but such responses alone may not be sufficient to prevent the negative effects of camouflage mismatch [21]. Dampening or disappearance of snowshoe hare population cycles could lead to reproductive failure of many avian and mammalian predators of the boreal forests, which may have cascading effects across trophic levels, with substantial consequences on the structure and function of boreal ecosystems. We note, however, that with changes in snow characteristics, the colour of the background habitat is also changing from mostly white to a mixture of brown and white from tree branches and fallen logs. Even at the present time in deep snow the colour of the background habitat is not uniformly white; for example, areas under spruce trees are often brown even with good snow conditions. Investigating how the background changes over winter with photos of hares made with continuously recording wildlife cameras from fixed camera sites could shed more precise light on winter camouflage in snowshoe hares inhabiting North American boreal forests. The background colour heterogeneity, insulation function and adaptive or plastic responses of coat colour to changing snow conditions may, in part, ameliorate the prediction that snowshoe hare survival will continue to decline under most climate change scenarios.

We do not know the exact reason why overwinter survival is affected by % white coat colour in this study. It could be the mismatch coupled with predation mortality as we have hypothesized here. This explanation will require details on the degree to which white or brown hares are mismatched with the changing timing of snow cover. Alternative explanations are that coat colour affects insulation, which in turn affects energetic requirements, foraging time and predator exposure, or that coat colour is an indicator of age, body weight, nutrient status, parasite loads and body condition, all of which could affect survival [49]. There is much left to do to understand the ecological effects of the changing timing of winter moulting of mammals in northern ecosystems.

## Data Availability

The datasets supporting this article have been uploaded as part of the electronic supplementary material [50] and also can be downloaded freely from: https://original-ufdc.uflib.ufl.edu/IR00011899/00001.
